# The Many Faces of Stress: Implications for Neuropsychiatric Disorders

**DOI:** 10.1155/2016/8389737

**Published:** 2016-03-17

**Authors:** Laura Musazzi, Jordan Marrocco

**Affiliations:** ^1^Laboratorio di Neuropsicofarmacologia e Neurogenomica Funzionale, Dipartimento di Scienze Farmacologiche e Biomolecolari and CEND, Università degli Studi di Milano, 20133 Milano, Italy; ^2^Harold and Margaret Milliken Hatch Laboratory of Neuroendocrinology, The Rockefeller University, New York, NY 10065, USA

Environmental stress is widely recognized as one of the main risk factors in neuropsychiatric diseases, including mood and anxiety disorders. Stress, with the ensuing activation of the hypothalamic-pituitary-adrenal axis, deeply affects neurotransmission and synaptic morphology in brain areas associated with behavioral responses and mental states. Clinical and preclinical studies have demonstrated that the impact of stressful life events on emotional and cognitive behaviors may vary depending on the nature of stress, its intensity or duration, and the time window of development during which stress exposure occurs (perinatally, adolescence, adulthood, or advanced age). The many-sided faces of stress also depend on brain region, sex, and individual differences.

When the stress response is efficient, it can induce adaptive neuroplasticity and improve cognition; however, when the stress response is overused, it can have toxic effects. A maladaptive stress response can lead to epigenetic changes associated with impaired brain functions and may ultimately trigger the development of neuropsychiatric disorders. Thus, the identification of neural mechanisms underlying resilience and vulnerability to stress is of crucial importance in understanding the pathophysiology of neuropsychiatric disorders and in developing improved treatments.

Four papers within the special issue deal with the long-term impact of early-life stress on neurotransmission, behavior, and coping strategies in the adulthood. In our review paper, we discuss the recent literature on the remodeling of excitatory neurotransmission and brain morphology, induced by acute and chronic stress in animal models. We also discuss that the integration between perinatal and late-life experiences may induce long-lasting consequences on neuronal excitatory transmission and morphology. We propose that reprogramming mechanisms may underlie the reorganization of excitatory neurotransmission in the short- and long-term response to stressful stimuli. The review by A. Ashokan et al. focuses on “stress inoculation,” suggesting that previous exposure to stress may improve coping strategies and stress resilience. The authors report experimental evidence indicating that moderate stressors, especially during early life, lead to enhanced health outcomes and stress resilience, while more severe stress may have deleterious consequences on health. G. Grigoryan and M. Segal contributed a review describing opposite functional and structural changes in dorsal versus ventral hippocampus induced by prenatal stress. This review summarizes the recent literature with emphasis on the importance of the septotemporal axis of the hippocampus in stress effects. Finally, an article by J. Maguire and I. Mody demonstrates that a mouse model of postpartum depression increases depressive- and anxiety-like behaviors in the offspring.

The issue contains three review papers highlighting stress as a major risk factor in the etiopathogenesis of stress-related disorders. First, D. Ness and P. Calabrese discuss the influence of stress on multiple memory systems and their contributions to the learning process leading to behavioral alterations. I. Negrón-Oyarzo et al. review the specific role of the prefrontal cortex in the maladaptive response to stress. The authors suggest that the impairment of functional connectivity within the prefrontal cortex induced by chronic stress has a mechanistic role in the etiopathogenesis of psychopathologies. The link between chronic stress and systemic illness (including neurological disorders, substance and alcohol abuse, neurodegenerative disorders, cardiovascular diseases, and gastrointestinal and metabolic disorders) is discussed by V. Duric et al. The authors emphasize that stress is a common risk factor in the pathophysiology of both psychiatric and systemic disorders.

Within the special issue, we have the honor to include a provocative review by E. R. de Kloet and M. L. Molendijk. The authors discuss the use of the forced-swim test as a reliable model for measuring depressive-like behavior. They report that the test is generally accepted to assess the antidepressant potential of drugs. However, rodents quickly activate coping strategies to adapt to the stressor, suggesting that the test could be used in the study of mechanisms of adaptation, rather than assessing depressive-like behavior.

Three research articles describe molecular and behavioral changes induced by stress in animal models of mood disorders. Y. Dwivedi and H. Zhang demonstrate reduced activation of the extracellular signal-regulated kinase 1/2 (ERK1/2) cascade in the brain of learned helplessness rats, highlighting the role of ERK1/2 signaling in stress vulnerability. The paper by A. Ieraci et al. describes behavioral deficits and changes in gene expression induced by social isolation in adulthood. In particular, the authors propose that increased anxious- and depressive-like behavior is associated with reduction of several neuroplasticity-related genes induced by social isolation stress. R. Molteni et al. show changes in activity-dependent transcription levels of Brain-Derived Neurotrophic Factor (BDNF) in the hippocampus of chronic mild stressed rats, a model that recapitulates the phenotypic hallmarks of depression.

Other research papers focus on both the immediate and delayed effects of different protocols of acute stress. D. Bonini et al. demonstrate that acute footshock stress induces time-dependent changes in glutamate receptor subunits in prefrontal and frontal cortex. Of note, this is consistent with stress-induced enhancement of glutamatergic synaptic transmission. H. A. Vecchiarelli et al. present a paper analyzing the time-dependent changes induced by acute stress. Here, selective time-dependent changes in enzymes belonging to the kynurenine metabolic pathway were found in corticolimbic areas of rats subjected to acute restraint stress. This suggests that the dysregulation of kynurenine metabolites may alter excitatory signaling. Another paper examines the long-term behavioral consequences of transient middle cerebral artery occlusion in rats. J. Kasahara et al. use a model of poststroke depression induced by transient ischemia. They demonstrate a spontaneous depressive-like phenotype 20 weeks after the induction of cerebral ischemia, which is associated with loss of granular neurons in the ipsilateral hippocampus, increased apoptosis, and changes in neurogenesis. Interestingly, these behavioral and cellular deficits were prevented by treatment with imipramine.

The present special issue also includes two papers focusing on the involvement of energy metabolism in the stress response. F. Jeanneteau and M. Arango-Lievano review the link between mitochondrial efficiency and synaptic activity. Mitochondria are key regulators of synaptic plasticity and changes in synaptic metabolism are involved in both adaptive and maladaptive mechanisms in response to stress. T. Larrieu et al. present evidence of molecular and morphological changes within the prefrontal cortex of mice fed with n-3 polyunsaturated fatty acid deficient diet. In particular, n-3 polyunsaturated fatty acid deficient diet induces alterations in the glucocorticoid receptor signaling pathway and reduces dendrite arborization.

Finally, we have the pleasure to include in this special issue a clinical research paper by C. Raymond et al., who report a pilot study on the effect of reading self-help books on stress reactivity and depression. The results demonstrate that consumers of growth-oriented self-help books showed higher cortisol reactivity to stress, whereas consumers of problem-focused self-help books manifested higher depressive symptomatology compared with nonconsumers. The authors suggest that reading self-help books might be associated with increased stress and/or mental disorders.

Collectively, this special issue includes 17 exciting papers that span diverse aspects of the multifaceted effects of stress, which involves either adaptive responses or increased vulnerability to neuropsychiatric disorders. We believe that this collection of manuscripts offers the latest insights into the molecular and neurochemical foundations of stress-related diseases, especially mood and anxiety disorders.

*Laura Musazzi* *Laura Musazzi* *Jordan Marrocco* *Jordan Marrocco*

